# Neuralgia—Tic Douloureux

**Published:** 1847-11

**Authors:** 


*Neuralgia—Tic Doloureux.* The following case, which occurred in the practice of R. H. Alkanett, M. D., &c., is important and interesting, inasmuch as it throws light upon at least one occasional cause of an acutely painful and obstinate affection, so frequently perplexing to the routine practitioner, who relies for its removal on the reckless administration of inordinate doses of the most powerful and highly deleterious narcotics. "A gentleman called on me a fortnight ago, suffering from a severe tic of the trifacial nerve, which branches up on the cheek and forehead, and the paroxysms observed something like an irregular periodicity. His appetite was good, and the functions of the stomach were opparently uninjured; the liver secreted a due quantity of bile; the bowels acted with regularity; and nothing could be detected, after the most minute search, to account for the agonizing pain in the peripheral expansions of the sentient nerve. As is my custom, I placed him under a preliminary course of active purgatives, and the pain in the face, and indeed all the symptoms, were aggravated to a considerable extent. Disregarding this temporary manifestation, I urged upon him the necessity of continuing the aperients until the full effect should be produced: and this morning he informed me that, in the middle of the night, he had a most copious evacuation; undetected scyabalæ, in large quantities, had been ejected from the bowels: he immediately experienced an inexpressible feeling of relief, and the facial tic has totally disappeared.—*Lancet.*

